# Comparative Efficacy, Safety, and Cost‐Utility of DPP‐4 Inhibitors and Metformin Combination Therapy in Type 2 Diabetes: A Systematic Review of Real‐World Clinical and Economic Outcomes

**DOI:** 10.1155/jdr/8464330

**Published:** 2026-04-02

**Authors:** Abdulgafar Olayiwola Jimoh, Shuaibu Abdullahi Hudu, Anas Ahmad Sabir, Adamu Ahmed Adamu, Nura Bello, Albashir Tahir, Nura Abubakar

**Affiliations:** ^1^ Department of Pharmacology and Therapeutics, Faculty of Basic Clinical Sciences, College of Health Sciences Usmanu Danfodiyo University, Sokoto, 840232, Sokoto State, Nigeria; ^2^ Center for Health Research, Northern Border University, Arar, 91431, Saudi Arabia, nbu.edu.sa; ^3^ Department of Microbiology, Faculty of Medicine, Northern Border University, Arar, 91431, Saudi Arabia, nbu.edu.sa; ^4^ Department of Internal Medicine, Faculty of Clinical Sciences, College of Health Sciences, Usmanu Danfodiyo University, Sokoto, Nigeria, udusok.edu.ng; ^5^ Department of Medicine, Usmanu Danfodiyo University Teaching Hospital, Sokoto, Nigeria, udusok.edu.ng; ^6^ Department of Pharmacology and Therapeutics, Faculty of Pharmaceutical Sciences, Ahmadu Bello University, Zaria, 810107, Kaduna State, Nigeria, abu.edu.ng; ^7^ Department of Pharmacology, Faculty of Basic Medical Sciences, Bauchi State University, Gadau, 751105, Nigeria, basug.edu.ng; ^8^ Department of Physiotherapy, Faculty of Basic Health Sciences, Zamfara State University, Talata Mafara, 892001, Nigeria

**Keywords:** cost-effectiveness, DPP-4 inhibitors, efficacy, metformin, type 2 diabetes mellitus

## Abstract

**Introduction:**

The management of type 2 diabetes with metformin as the first‐line therapy has long been established. However, combination therapy of metformin and other oral antidiabetics became necessary to achieve optimal glycemic targets. Recently, the rising cost of these combinations poses a challenge for the healthcare system and patients, particularly in low‐ and middle‐income settings, highlighting the need to balance clinical benefits with economic considerations to ensure access to treatment while maintaining sustainability in patient care. This review aims to compare the efficacy, safety, and cost‐effectiveness of DPP‐4 inhibitors with metformin/metformin with other combinations and metformin alone.

**Methods:**

A literature search was performed through databases including PubMed, Scopus, Cochrane, clinicaltrials.gov, and Google Scholar using specific keywords on “type 2 diabetes mellitus management,” “metformin,” “DPP‐4 inhibitors,” “safety,” and “efficacy.” The retrieved studies were screened and selected according to eligibility criteria, followed by data extraction and critical appraisal. The extracted data were synthesized and reported according to the PRISMA guidelines.

**Results:**

Thirty‐five eligible studies were included in the review. From the studies, oral antidiabetic options apart from DPP‐4 inhibitors commonly combined with metformin, either as free or fixed‐dose combinations, include SGLT‐2 inhibitors, sulfonylureas, GLP‐1 receptor agonists, insulin, and thiazolidinediones (TZDs). The efficacy of this drug combination is comparable to that of metformin monotherapy, which is more cost‐effective, especially at the beginning of treatment. Where metformin monotherapy fails, the efficacy of an add‐on therapy as a second line depends on the specific target and individual patient differences, and even triple therapy may be recommended for some individuals. The cost‐effectiveness of each combination depended on the cost‐effectiveness model used in the assessment and the nature of the healthcare setting.

**Discussion:**

DPP‐4 inhibitors/metformin demonstrate significant HbA1c reduction, but their low cost‐effectiveness hinders patient adherence compared to metformin monotherapy, free drugs, or other combinations. For that, initiating therapy with cost‐effective metformin alone is recommended. Manufacturer‐funded trials highlight a potential bias, necessitating independent research validations.

## 1. Introduction

Type 2 diabetes mellitus (T2DM) is a chronic metabolic disorder characterized by persistent hyperglycemia due to insulin resistance and insufficient insulin production secondary to progressive *β*‐cell dysfunction [1]. It is the most common form of diabetes, accounting for over 90% of cases worldwide, and is increasingly being diagnosed in children and adolescents, mainly due to the growing obesity epidemic [2]. T2DM develops when the body’s cells no longer respond effectively to insulin, the hormone responsible for glucose uptake. Initially, the pancreas compensates by producing more insulin, but over time, this mechanism fails, leading to sustained high blood glucose levels (hyperglycemia) and increased risk of microvascular and macrovascular complications [3]. Managing T2DM involves controlling blood sugar levels to prevent complications, which is achieved through a combination of medications (oral hypoglycemics) and lifestyle changes. Among these, metformin is widely recognized as the first‐line treatment for its proven efficacy in lowering blood glucose levels, its favorable safety profile, and its additional benefits in weight management and cardiovascular health [4]. However, when metformin alone is insufficient to achieve glycemic targets and the disease progresses, combination therapy becomes necessary. Among these, a fixed‐dose combination of DPP‐4 inhibitors and metformin, such as vildagliptin–metformin (Galvus–Met) and sitagliptin–metformin (Trivia–Met), is often recommended and has gained widespread use. The dual‐action mechanism of such combinations improves glucose regulation through complementary pathways and has been associated with significant reductions in HbA1c levels [5].

Although fixed‐dose combinations (FDCs), such as DPP‐4 inhibitors + metformin, are convenient, simplifying treatment regimens and potentially improving adherence, their high production costs often translate into exorbitant market prices. This makes them inaccessible to many patients, especially in low‐income settings where access to healthcare and insurance coverage is limited [6]. The high cost of such therapies often leads to noncompliance among patients, resulting in poor glycemic control and the worsening of T2DM, with complications that could have been avoided with more affordable medications [7]. The need to apply pharmacoeconomic principles in diabetes management cannot be overemphasized. Pharmacoeconomics helps assess the value of drugs by comparing their costs to their outcomes, such as effectiveness, quality of life, and patient satisfaction [8]. This approach ensures that the best treatment options are available at a cost that the patient can afford. Research has shown that vildagliptin–metformin combinations, such as Galvus–Met, provide significant benefits for patients by addressing multiple pathways of glucose regulation [9, 10]. These combinations can also reduce the risk of costly complications, such as cardiovascular disease and hospitalizations [11].

However, the cost‐effectiveness of branded medications versus generic alternatives often depends on patient access and insurance coverage [12]. This makes it essential to evaluate whether the additional benefits of DPP‐4 inhibitors + metformin justify their higher cost compared to generic metformin and other combinations. Although numerous clinical trials, systematic reviews, and meta‐analyses have established the efficacy and safety of DPP‐4 inhibitor‐metformin combinations, significant gaps remain in the comparative evaluation of their cost‐effectiveness and real‐world affordability, particularly in low‐ and middle‐income settings. By comparing these therapies, this systematic review aims to address this knowledge gap and comprehensively assess the efficacy, safety, and cost‐effectiveness of DPP‐4 inhibitor‐metformin combinations with metformin monotherapy and other antidiabetic combinations. By synthesizing evidence across these domains, this review seeks to provide a clear, clinically relevant assessment of whether the additional benefits of DPP‐4 inhibitor‐based combinations justify their higher costs. The findings will guide healthcare providers in selecting treatments that balance efficacy, affordability, and patient needs, ultimately improving diabetes management for diverse populations and identifying alternative options when a particular treatment is unaffordable.

## 2. Methods

### 2.1. Data Sources and Search Strategy

The articles were searched in PubMed, Google Scholar, Scopus, the Cochrane Library, clinicaltrials.org, and other databases. The search was conducted in December 2025, and the included articles were published between 2008 and 2024. Keywords including “Type 2 Diabetes Mellitus,” “adult‐onset diabetes,” “Non‐Insulin‐Dependent Diabetes Mellitus,” “DPP‐4 inhibitor,” “Glucophage,” “vildagliptin,” “metformin,” and “cost‐effectiveness” were part of the search strategy. The terms were combined and searched using Boolean operators. All studies and research included in this systematic review were from published data. The search results were screened, and relevant studies were selected according to the inclusion and exclusion criteria.

#### 2.1.1. Inclusion Criteria

Studies that investigate cost‐effectiveness, QALYs, safety, and efficacy of metformin and DPP‐4 inhibitors compared to other combinations in type 2 diabetes. Publication date, studies measuring glycemic control (HbA1c) or adherence/cost‐effectiveness. Cost‐effectiveness studies, randomized controlled trials (RCTs), cohort studies, and systematic review and meta‐analysis studies.

#### 2.1.2. Exclusion Criteria

Studies without cost‐effectiveness analysis, studies focused on type 1 diabetes, studies with pediatric populations (unless directly relevant), studies not published in English, studies with incomplete or unclear data, low‐quality studies (e.g., small sample size, poor study design, and biased sampling), and articles not available in full text or unpublished manuscripts.

### 2.2. Screening Process

Search results were downloaded into the reference manager, and duplicates were removed. Two investigators independently screened titles and abstracts to identify relevant studies that met the eligibility criteria. Studies that could confidently be judged as ineligible based on title/abstract alone were excluded. Full texts were screened when a decision on inclusion/exclusion could not be reached through an assessment of the title and abstract alone.

### 2.3. Data Extraction and Quality Assessment

**Figure 1:**
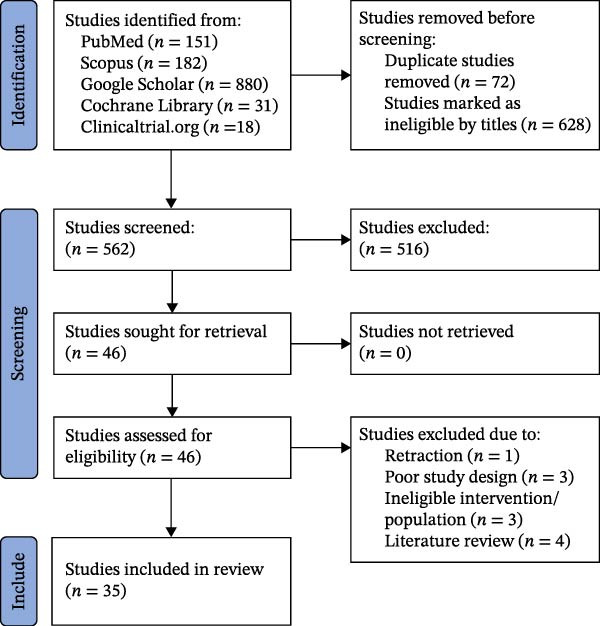
Preferred reporting items for systematic reviews and meta‐analyses flowchart of database search.

The data extracted were synthesized and reported according to the PRISMA guideline (Figure 1) from eligible studies using a predefined data extraction sheet designed to record the following: author’s name, publication year, country, and study design (i.e., RCTs, cohort studies, cost‐effectiveness studies, and systematic reviews). Population/demographics, comparison group, intervention, pharmacoeconomics, and outcome status were reported.

Three of the authors undertook the data extraction, which was checked against the original paper by an independent reviewer. Disagreements were resolved by discussion or through arbitration between the independent reviewers. Each eligible study was read by three reviewers and assigned a quality assessment score based on criteria adapted from the Critical Appraisal Skills Programme (CASP) tools. Studies received one point for each criterion they were judged to have met successfully, yielding a potential maximum score of points for study quality.

### 2.4. Risk of Bias Assessment

The risk of bias of included studies was assessed using the Cochrane Risk of Bias Assessment Tool (http://handbook.cochrane.org/) for RCTs and the CASP tools for nonrandomized studies of interventions (e.g., cohort or case‐control studies), systematic reviews, and economic model studies. At least three reviewers reviewed all studies, and any disagreements were resolved by involving another independent reviewer.

## 3. Result

### 3.1. Overview of Included Studies

Thirty‐five studies were selected and included in the review based on the inclusion and exclusion criteria. The studies include cohort studies (6 studies), as shown in Table 1; clinical trials (6 studies), as shown in Table 2; cost‐effectiveness analyses (13 studies); and systematic reviews (9 studies), as shown in Table 3. The most significant number of studies was from Europe, with 16 studies (46%) conducted in countries such as the UK, France, Germany, Spain, and Sweden, primarily focused on cost‐effectiveness analyses (Table 4), cohort studies, and systematic reviews. Nine studies (26%) were from North America, with the highest number from the USA and Canada, which involved cost‐effectiveness analyses and systematic reviews. Asia accounts for seven studies (20%), primarily from China, India, Japan, and South Korea, focusing on clinical trials, cohort studies, and cost‐effectiveness analyses. South America is represented by three studies (9%) from Ecuador, Colombia, and Brazil, mainly on cost‐effectiveness analyses. Finally, Africa has only one study (3%) from Nigeria, which is a clinical trial. The studies also compare various interventions for T2DM in terms of efficacy, safety, and cost‐effectiveness, measured using multiple parameters.

**Table 1:** Cohort studies.

S/N	Reference	Country	Intervention	Comparison	Assessment parameters
1	[13]	India	Metformin + teneligliptin	High‐dose metformin	FBS, PPBS, and glycemic control
2	[14]	Spain	Metformin + DPP‐4 inhibitors	Metformin + other oral antidiabetic drugs	Compliance, persistence, HbA1*c* < 7%, hypoglycemia, and healthcare costs
3	[15]	Spain	Metformin + DPP‐4 inhibitors	Metformin + sulfonylureas	HbA1c, compliance, cardiovascular events, hypoglycemia, and healthcare costs
4	[16]	Japan	DPP‐4 inhibitors	Metformin	HbA1c and drug costs
5	[17]	USA	Sitagliptin + metformin	Sulfonylureas + metformin	Adherence and out‐of‐pocket costs
6	[18]	USA	DPP‐4 inhibitors + metformin	Sulfonylureas + metformin	Adherence, persistence, and progression to insulin

**Table 2:** Clinical trials.

S/N	Reference	Country	Intervention	Comparison	Assessment parameters
1	[19]	France	Fixed‐dose vildagliptin/metformin	Vildagliptin and metformin separately	Bioequivalence and adverse effects
2	[20]	South Korea	Triple therapy (metformin, dapagliflozin, saxagliptin)	Stepwise add‐on therapy	HbA1c, hypoglycemia, adherence, and adverse effects
3	[21]	Nigeria	Vildagliptin + metformin	Metformin + glibenclamide	HbA1c, adverse effects, BMI, and hypoglycemia
4	[22]	India	Metformin + teneligliptin	Metformin + glimepiride	HbA1c, fasting blood glucose, and adverse effects
5	[23]	UK	Vildagliptin	Sulfonylureas	HbA1c, adherence, and hypoglycemia
6	[24]	India	Sulfonylurea + metformin	Glitazones/DPP‐4 inhibitors + metformin	HbA1c, fasting plasma glucose, and cost‐effectiveness

**Table 3:** Systematic reviews.

S/N	Reference	Country	Intervention	Comparison	Assessment parameters
1	[25]	USA	New antidiabetic medications	NA	Cost per QALY
2	[26]	Canada	DPP‐4 inhibitors	Sulfonylureas	HbA1c, body weight, and hypoglycemia
3	[27]	China	DPP‐4 inhibitors	Metformin, sulfonylureas, and others	Cost‐effectiveness
4	[28]	Multiple regions	SGLT2i, GLP‐1RA, DPP‐4i + metformin	Other antidiabetics	Cost‐effectiveness
5	[29]	Multiple regions	DPP‐4 inhibitors	Sulfonylureas, GLP‐1RA, insulin	Cost‐effectiveness
6	[30]	Multiple regions	DPP‐4 inhibitors	Other oral antidiabetics	Cost‐effectiveness
7	[31]	Multiple regions	Monotherapies and combinations	Various	HbA1c, cardiovascular mortality, body weight, and hypoglycemia
8	[32]	Sweden	GLP‐1 agonists, DPP‐4 inhibitors	NPH insulin	Incremental cost‐effectiveness ratios (ICERs)
9	[33]	Colombia	Dapagliflozin + metformin	DPP‐4 inhibitors	ICER and QALYs

**Table 4:** Cost‐effectiveness analyses.

S/N	Reference	Country	Intervention	Comparison	Assessment parameters
1	[34]	Sweden	Saxagliptin + metformin	Sulfonylurea + metformin	ICER, HbA1c, hypoglycemia, and weight changes
2	[22]	UK	Various treatment escalation strategies	Other combinations	HbA1c, costs, and QALYs
3	[35]	USA	Metformin + DPP‐4 inhibitors	Metformin + sulfonylureas	ICER, hypoglycemia, and costs
4	[36]	UK	Dapagliflozin + metformin	DPP‐4 inhibitors	QALYs and ICER
5	[37]	Ecuador	Metformin + sitagliptin	Metformin + glibenclamide	ICER
6	[38]	Greece	Dapagliflozin + metformin	Sulfonylurea, DPP‐4 inhibitors	ICER
7	[39]	Germany	Saxagliptin + metformin	Sulfonylurea	QALYs, ICER, and hypoglycemia
8	[40]	China	Saxagliptin + metformin	Glimepiride + metformin	ICER and costs
9	[41]	USA	Liraglutide + metformin	Sitagliptin	ICER and QALYs
10	[42]	Portugal	Metformin + vildagliptin	Metformin + sulfonylureas	ICER
11	[32]	Sweden	GLP‐1 agonists, DPP‐4 inhibitors	NPH insulin	Incremental cost‐effectiveness ratios
12	[29]	Multiple	DPP‐4 inhibitors	Sulfonylureas, GLP‐1 RA	ICER and cost‐effectiveness
13	[33]	Colombia	Dapagliflozin + metformin	DPP‐4 inhibitors	ICER and QALYs

### 3.2. Result of the Critical Appraisal of Studies

The included systematic reviews were thorough in addressing the focused questions and often conducted comprehensive searches for relevant studies. They were notable in assessing quality, combining results reasonably, and examining important outcomes such as HbA1c reduction, cost per QALY, and adverse effects. However, the completeness of some reviews was compromised by narrow search strategies or language restrictions, potentially missing key studies. Variability in applicability to local populations also emerged, as healthcare systems and economic contexts often differed significantly from those in which the reviews were conducted.

The included cohort studies provided valuable insights into relevant issues, including the effectiveness of diabetes treatments on glycemic control, adherence, and healthcare costs. These studies were meticulous in outcome measurement and often accounted for confounding factors through thoughtful design and analysis. Nevertheless, concerns about selection bias arose when recruitment methods were not well‐detailed, and incomplete follow‐up data or dropout rates occasionally weakened the reliability of findings. Despite these challenges, the evidence generated is frequently aligned with existing research, contributing to robust clinical and economic understandings.

The included clinical trials were methodologically rigorous, primarily by employing robust randomization procedures and achieving comparable baseline characteristics across study groups. Comprehensive reporting of intervention effects, encompassing both primary outcomes such as HbA1c and secondary measures such as weight changes or hypoglycemia, was presented. In contrast, the implementation of blinding was inconsistent, especially in open‐label trials, creating a potential for outcome bias. Furthermore, while the benefits of experimental interventions were well‐documented, discussions of their cost implications and feasibility in lower‐resource settings were often insufficient. These trials nonetheless provided critical evidence on the efficacy and safety of interventions to support clinical decision‐making.

Economic evaluations were notable for their clarity, specificity of research questions, and detailed comparative analyses of contending alternatives. By employing established models such as ICER and conducting sensitivity analyses, they strengthened the robustness of the findings and effectively highlighted cost‐effectiveness. However, reliance on cost structures and healthcare practices specific to particular countries sometimes limited the generalizability of the results. Despite providing valuable insights into cost‐effective strategies, translating and applying the findings of these evaluations to diverse populations and healthcare systems may prove to be a significant challenge.

### 3.3. Metformin as a First‐Line Therapy in the Management of Type 2 Diabetes

The role of metformin as a first‐line drug in type 2 diabetes is supported by extensive evidence emphasizing its cost‐effectiveness, glycemic control efficacy, and broader health benefits. Its affordability and favorable economic profile make it an optimal choice for initial therapy [22], underscoring metformin’s ability to delay the need for more expensive combination therapies while maintaining effective glycemic control. Cost‐effectiveness analyses further confirmed that metformin‐based regimens achieve significant clinical outcomes at an acceptable cost per quality‐adjusted life year (QALY) [34, 42]. This economic advantage is particularly impactful in low‐resource settings, where treatment affordability directly influences adherence and outcomes [43].

The glycemic efficacy of metformin is strong, with reductions in HbA1c often reaching 1%–1.5%, and achieving glycemic targets comparable to combination therapies over extended durations, demonstrating its effectiveness as a monotherapy [13]. Combination regimens initiated with metformin offer enhanced durability of glycemic control, delaying the need for further therapeutic escalation [20]. Furthermore, metformin is one of the few antidiabetic agents proven to reduce macrovascular complications, with studies reporting reductions in cardiovascular mortality and major adverse cardiovascular events (MACE) [31]. These findings establish metformin as both a glucose‐lowering and a cardioprotective agent [44].

Gastrointestinal side effects are common with metformin, but they are transient and manageable, particularly with extended‐release formulations (metformin XR), which improve tolerability and adherence. Higher adherence to metformin‐based regimens directly correlates with better long‐term glycemic and cardiovascular outcomes [31]. Its versatility extends to conditions such as polycystic ovary syndrome (PCOS) and insulin‐resistant states, showcasing its broader therapeutic potential [45].

However, despite their strengths, newer agents like SGLT‐2 inhibitors and GLP‐1 receptor agonists (GLP‐1 RAs) offer additional cardiovascular and renal benefits in high‐risk populations. These therapies are often integrated as second‐line options following metformin, emphasizing its indispensable role as the foundation of diabetes care. The benefits of metformin can be maximized while still addressing specific clinical needs by tailoring therapy to individual patient profiles.

### 3.4. Comparative Analysis of Metformin‐Based Combination Therapies

#### 3.4.1. DPP‐4 Inhibitors + Metformin Vs. Sulfonylureas (SUs) + Metformin

Exploring the differences between DPP‐4 inhibitors and SUs, both in combination with metformin, reveals unique strengths and limitations of each combination in managing type 2 diabetes. DPP‐4 inhibitors demonstrate moderate but consistent reductions in HbA1c, typically between 0.44% and 0.67% over one to 2 years [16, 42]. In contrast, SUs often achieve slightly better initial reductions in HbA1c. For instance, reductions of up to −1.19% have been observed in shorter‐term studies, particularly when glimepiride is used alongside metformin [24]. However, these benefits are often less durable over the long term due to a higher likelihood of treatment failure and progression to insulin [18]. DPP‐4 inhibitors and SUs differ significantly in their effects on weight and cardiovascular outcomes. DPP‐4 inhibitors are largely weight‐neutral or may even lead to a slight weight loss of around 0.5–1.0 kg [34, 42]. In addition, they reduce the risk of cardiovascular events compared to SUs, with Sicras‐Mainar et al. [15] reporting lower rates of cardiovascular events (3.7% vs. 6.4%) in patients treated with DPP‐4 inhibitors. SUs, in contrast, are frequently associated with weight gain, often averaging 1–2 kg, as well as a higher risk of cardiovascular complications, especially in patients with preexisting cardiovascular conditions [26, 40]. However, in line with the ADA Standards of Care in Diabetes‐2025, DPP‐4 inhibitors are considered to have a generally neutral cardiovascular profile, with cardiovascular safety demonstrated through noninferiority rather than cardioprotective benefit [46]. Therefore, the observed differences in cardiovascular event rates likely reflect differences in safety rather than a class‐wide cardiovascular risk‐reducing effect.

One of the most significant distinctions between these two combinations is their risk of hypoglycemia. DPP‐4 inhibitors are known for their low risk of hypoglycemia, making them particularly attractive for patients who are prone to or fear this complication [15, 23]. SUs, however, have a well‐documented association with hypoglycemia, with rates as high as 40.4% reported in some studies, particularly for older agents like glibenclamide [15]. This elevated risk often necessitates closer monitoring and may deter some patients from continuing treatment. Cost considerations may also play a significant role in the decision between the two options. DPP‐4 inhibitors are typically more expensive upfront but can be more cost‐effective in the long run due to their reduced risks of hypoglycemia and cardiovascular events, according to cost‐effective models in some countries. For example, Sicras‐Mainar et al. [15] reported that total healthcare costs were lower for DPP‐4 inhibitors (€2341 vs. €2512) over 2 years, owing to fewer complications and hospitalizations. Sulfonylurea combinations are generally more affordable in terms of direct drug costs but may often incur higher long‐term costs associated with managing hypoglycemia and other adverse events [35].

Adherence and persistence tend to be higher with DPP‐4 inhibitors in some settings, due to their tolerability and ease of use. A study by Peng et al. [18] reported adherence rates exceeding 65% and persistence rates of around 52.5% after 1 year. At the same time, SUs, in comparison, are associated with lower adherence due to side effects like weight gain and hypoglycemia, as well as a higher likelihood of treatment escalation [18]. These factors might lead to poorer long‐term outcomes for sulfonylurea‐based therapies. Regionally, preferences for these treatments vary. In high‐income countries, DPP‐4 inhibitor combinations are often favored for their overall safety and tolerability, despite their higher cost [35]. In middle‐income settings, such as India and Ecuador, SUs remain an indispensable treatment due to their affordability and accessibility. However, DPP‐4 inhibitors are gaining traction for their safety profile and longer‐term cost‐effectiveness [22, 37].

DPP‐4 inhibitors combined with metformin offer a safer and more tolerable alternative to SUs, particularly for patients at higher risk of hypoglycemia or cardiovascular complications. SUs may provide slightly greater initial reductions in HbA1c and are more cost‐effective in the short term. Still, their long‐term disadvantages often make DPP‐4 inhibitors the better choice for sustained diabetes management. These findings therefore stress the importance of tailoring treatment plans to individual patient needs and healthcare system constraints.

#### 3.4.2. DPP‐4 Inhibitors + Metformin Vs. SGLT‐2 Inhibitors + Metformin

The comparison between DPP‐4 inhibitors and SGLT‐2 inhibitors, both combined with metformin, highlights some crucial differences in their effectiveness and overall benefits for managing type 2 diabetes. DPP‐4 inhibitors are effective at lowering HbA1c, with reductions of 0.44%−0.57% over 1 year, as reported in studies using vildagliptin combined with metformin [34, 42]. Another survey by Poornima et al. [13] found that patients using a combination of metformin and teneligliptin achieved a fasting blood glucose (FBS) of 125.83 mg/dL, compared with 125.77 mg/dL with metformin alone. Alternatively, SGLT‐2 inhibitors appear to have an advantage in glycemic control, as dapagliflozin and metformin were shown to reduce HbA1c by up to 1.4%, offering a clear improvement compared to DPP‐4 inhibitors [33, 40]. When it comes to weight management and cardiovascular outcomes, DPP‐4 inhibitors are generally weight‐neutral or may even cause slight weight loss of about 0.5–1.0 kg [34, 35]. They also show fewer cardiovascular events than SUs, though their benefits are not as pronounced as those of SGLT‐2 inhibitors [15]. SGLT‐2 inhibitors are therefore preferred when cardiovascular risk reduction is a primary therapeutic objective, since DPP‐4 inhibitors are regarded as cardiovascular‐benefit neutral, with no confirmed reduction in major cardiovascular events [46]. Meanwhile, SGLT‐2 inhibitors are known to achieve more substantial weight loss, typically between 1 and 2 kg, as reported in many studies [38]. They also play a vital role in reducing cardiovascular risks, particularly for patients with established cardiovascular disease. It was even noted that SGLT‐2 inhibitors significantly reduce cardiovascular events, making them a standout option for at‐risk patients [28].

In terms of safety, DPP‐4 inhibitors are effective in reducing the risk of hypoglycemia, with a much lower risk than SUs [23, 27]. SGLT‐2 inhibitors are also rarely associated with hypoglycemia, except when used alongside insulin or SUs [28]. Looking at cost‐effectiveness, DPP‐4 inhibitors hold their own against SUs, with incremental cost‐effectiveness ratios (ICERs) of around €9072/QALY [42]. However, SGLT‐2 inhibitors tend to be even more cost‐effective, with ICERs as low as $1964.80/QALY in some studies, mainly due to their ability to reduce complications and healthcare costs over time [33]. Adherence and persistence also play a crucial role in determining the practicality of these treatments. Patients on DPP‐4 inhibitors tend to stick to their treatment better, with adherence rates over 65% and persistence between 52.5% and 70.3%, likely due to their tolerability and convenient oral administration [15, 18]. SGLT‐2 inhibitors, though slightly less consistent in adherence, are valued for additional benefits such as weight reduction and cardiovascular protection, which make them harder to discontinue [28, 38]. However, typical side effects, such as genitourinary infections, can lead to discontinuation.

Geography may also influence the choice between these two drug classes. In high‐income settings such as Europe and the US, SGLT‐2 inhibitors are often preferred for their strong cost‐effectiveness and cardiovascular advantages [28, 36]. In middle‐income countries such as India and Colombia, DPP‐4 inhibitors remain widely used due to their affordability and reasonable efficacy. However, they fall slightly short in cardiovascular benefits compared to SGLT‐2 inhibitors [22, 37].

Both DPP‐4 inhibitors and SGLT‐2 inhibitors, when combined with metformin, are effective for managing blood sugar, with SGLT‐2 inhibitors standing out for their additional benefits, such as weight loss, cardiovascular protection, and overall cost‐effectiveness. For that, the decision to use one over the other depends on the individual patient’s characteristics, risk factors, and access to healthcare resources. The studies in this review reinforce these points, providing strong evidence to guide clinical decisions.

#### 3.4.3. DPP‐4 Inhibitors + Metformin Vs. GLP‐1 RAs + Metformin

Comparing DPP‐4 inhibitors and GLP‐1 RAs in combination with metformin for managing type 2 diabetes revealed notable differences. DPP‐4 inhibitors are well‐regarded for their ability to lower HbA1c levels, with reductions typically ranging from 0.44% to 0.67% over one to 2 years [16, 42]. While these results are effective, GLP‐1 RAs often achieve superior glycemic control. For example, studies involving liraglutide with metformin reported reductions in HbA1c of 1.29% to 1.51%, making them particularly effective for patients with higher blood sugar levels [28, 41]. On examining weight and cardiovascular outcomes, DPP‐4 inhibitors tend to be neutral or slightly beneficial for weight management, with mild reductions of 0.5–1.0 kg reported [16, 34].

Additionally, they offer modest cardiovascular benefits, particularly through their low risk of hypoglycemia and lack of adverse effects on lipid profiles [21]. However, they are primarily selected for glycemic control and tolerability rather than cardiovascular risk reduction. However, GLP‐1 RAs take these benefits further by delivering substantial weight loss, often in the range of 2–3 kg, as highlighted in several studies, including liraglutide trials that consistently showed weight reductions around 2.2 kg [26]. Their impact on cardiovascular health is also more pronounced, with significant reductions in cardiovascular events reported, particularly in high‐risk patients [28, 32].

Both classes of drugs have a low risk of hypoglycemia, though their cost profiles differ. Despite being more cost‐effective than SUs and insulin, DPP‐4 inhibitors are often outperformed by GLP‐1 RAs in overall value, as noted in some studies. For example, GLP‐1 RAs incur higher upfront costs but also provide greater QALYs and are considered cost‐effective in high‐income settings. Also, the ICERs for GLP‐1 RAs compared to DPP‐4 inhibitors were SEK 353,172/QALY, highlighting the tradeoff between cost and long‐term clinical benefits [32]. Patient adherence and persistence rates also differ between these two drug classes. With their convenient oral administration and good tolerability, DPP‐4 inhibitors enjoy higher adherence rates, often exceeding 65%, and persistence rates of around 52.5% over a year [14, 18]. GLP‐1 RAs, on the other hand, face some challenges in adherence due to being injectable and causing side effects like nausea and vomiting, which can discourage some patients [41]. However, for patients motivated by the significant weight loss and cardiovascular benefits, GLP‐1 RAs remain a compelling choice.

Preferences for these treatments also vary by region. In high‐income countries such as the US and Europe, GLP‐1 RAs are increasingly preferred, especially for patients with obesity or cardiovascular conditions, owing to their superior outcomes in weight loss and cardiovascular protection [28, 32]. Meanwhile, in middle‐income settings, DPP‐4 inhibitors are often favored due to their affordability and satisfactory efficacy. GLP‐1 RAs are typically reserved for selected patient groups who can manage the higher costs [37]. Ultimately, the decision between DPP‐4 inhibitors and GLP‐1 RAs combined with metformin depends on the patient’s clinical needs, preferences, and healthcare resources. GLP‐1 RAs excel in areas like glycemic control, weight loss, and cardiovascular protection. At the same time, DPP‐4 inhibitors remain a practical and cost‐effective alternative, particularly for patients who prefer oral medications or have lower cardiovascular risk. These findings, supported by insights from the reviewed studies, underscore the strengths and limitations of each option, offering clear guidance for individualized diabetes management.

### 3.5. Comprehensive Comparison of Metformin‐Based Treatment Regimens for Type 2 Diabetes

The table below (Table 5) provides a concise overview of the clinical outcomes, adherence, safety, weight effects, and cost‐effectiveness of the various treatment regimens for type 2 diabetes mellitus based on findings from this systematic review.

**Table 5:** Comparative overview of metformin‐based combinations for type 2 diabetes across key parameters.

Parameter	Metformin monotherapy	Metformin + sulfonylureas	Metformin + DPP‐4 inhibitors	Metformin + SGLT‐2 inhibitors	Metformin + GLP‐1 RAs	Fixed‐dose DPP‐4/metformin
HbA1c reduction	1%–1.5%	Up to −1.19%	0.44%–0.67%	Up to −1.4%	1.29%–1.51%	Bioequivalence to individual components
Adherence	High	Moderate	High	Moderate to high	Moderate (due to injectability)	High
Hypoglycemia risk	Low	High	Low	Low	Low	Low
Weight impact	Weight‐neutral	Weight gain (1–2 kg)	Weight‐neutral or slight loss (0.5–1 kg)	Significant weight loss (1–2 kg)	Substantial weight loss (2–3 kg)	Weight‐neutral or slight loss
Cardiovascular benefits	Neutral	Minimal	Neutral	Significant reduction in cardiovascular events	Significant reduction in cardiovascular and renal events	Neutral
Fasting blood glucose (FBS)	Moderate reductions	Significant reduction	Moderate reductions	Significant reductions	Significant reductions	Moderate reductions
Postprandial blood glucose (PPBS)	Moderate reductions	Significant reduction	Moderate reductions	Significant reductions	Significant reductions	Moderate reductions
Cost‐effectiveness	Cost‐effective as a first‐line therapy	Affordable upfront; costly long‐term due to hypoglycemia	High upfront costs; long‐term savings from fewer complications	Highly cost‐effective, particularly in high‐risk patients	Cost‐effective in high‐income settings but expensive upfront	Often unaffordable in low‐income settings

### 3.6. The Cost and Affordability of DPP‐4 Inhibitors for Managing T2DM in Low‐Income Settings

The cost and affordability of combining DPP‐4 inhibitors with metformin pose significant challenges in low‐income settings, particularly where healthcare subsidies are limited. Although these FDCs deliver excellent clinical outcomes, their high costs often make them inaccessible to many patients in resource‐constrained environments. This affordability gap is evident when compared with more cost‐effective alternatives such as SUs, despite the clinical superiority of DPP‐4 inhibitors. DPP‐4 inhibitors consistently show better adherence, metabolic control, and lower rates of adverse events like hypoglycemia, as hinted by studies in Spain, which found that metformin–DPP‐4 combinations reduced overall healthcare costs (€2341 vs. €2512) compared to SUs, owing to better adherence rates (70.3% vs. 60.6%) and fewer hypoglycemic events (13.9% vs. 40.4%) [14]. However, this cost‐saving potential is often outshone by the upfront drug acquisition costs. In Japan, for example, annual costs for DPP‐4 inhibitors were $458.7, compared to $273.3 for baseline therapies, a disparity that is magnified in low‐resource settings without subsidies [16]. Studies in middle‐income countries further highlight the affordability barriers. In Ecuador, sitagliptin combined with metformin was cost‐effective in the public health system, with an ICER of −$1621.85 per QALY. However, in semi‐private settings, the ICER rose dramatically to −$19,131.61 per QALY, making it unaffordable [37]. Similarly, a study in India demonstrated that glimepiride–metformin combinations were far more cost‐effective than teneligliptin–metformin, reinforcing the reliance on SUs in low‐income environments [22]. High‐income settings offer valuable insights for examining sensitivity analyses and determining cost‐effectiveness thresholds. In Portugal, the ICER for vildagliptin–metformin was €9072 per QALY, deemed cost‐effective within the Portuguese healthcare system but likely unaffordable in low‐income settings without robust subsidies [42]. At the same time, an evaluation of saxagliptin with metformin in Sweden reported incremental costs of SEK 9484 per QALY compared with SUs, which were cost‐effective under Swedish thresholds but far beyond the budgets of underfunded healthcare systems [34].

The disparity in costs is further evident in Nigeria, where vildagliptin–metformin demonstrated superior clinical outcomes compared with metformin–glibenclamide, including reduced weight gain and fewer hypoglycemic episodes. However, its adoption was hampered by limited healthcare financing and the high cost of DPP‐4 inhibitors [21]. In the United States, over 25 years, the total cost of DPP‐4/metformin combinations was $18,853, compared with $7004 for sulfonylurea/metformin combinations, even though DPP‐4 inhibitors yielded greater life‐year gains and fewer complications [35]. Studies also suggest that better glycemic outcomes and tolerability associated with DPP‐4 inhibitors might imply cost‐effectiveness in specific contexts, even in the absence of robust pharmacoeconomic data [13]. However, their high upfront costs remain a significant barrier to accessibility in many low‐income regions. As noted by Suraj et al. [24], SUs continue to dominate in these settings due to their lower costs, despite higher risks of adverse events and less favorable long‐term outcomes.

As DPP‐4 inhibitors combined with metformin offer clear clinical and long‐term economic benefits, their high cost limits their utility in low‐income settings. Without substantial subsidies, price reductions, or systemic interventions, these combinations will struggle to replace cheaper alternatives like SUs. Sensitivity analyses from higher‐cost environments highlight the significant financial thresholds required to justify their use, making accessibility in underfunded healthcare systems a persistent challenge.

## 4. Discussion

In this systematic review, we provide valuable insights into the clinical outcomes, safety, and economic value of DPP‐4 inhibitors combined with metformin compared to other treatment options for managing Type 2 Diabetes Mellitus (T2DM). DPP‐4/metformin combinations consistently demonstrated moderate efficacy, achieving HbA1c reductions of 0.44% to 0.67% [34, 42]. These combinations also showed a favorable safety profile, characterized by a low risk of hypoglycemia and weight neutrality, making them suitable for patients requiring safe and tolerable long‐term therapies [18]. However, from an affordability perspective, these combinations pose a significant concern, especially in low‐income settings with limited access to healthcare subsidies. Evidence from studies conducted in Ecuador and Nigeria indicates that even in regions where cost‐effectiveness has been demonstrated under subsidized systems, these treatments often remain inaccessible in semi‐private or uninsured settings [21, 37]. This raises important questions about the practical applicability of these therapies in resource‐limited environments.

In settings where healthcare budgets are constrained, the high cost of DPP‐4/metformin FDCs serves as a substantial barrier to their accessibility and widespread use. Although the fixed‐dose format improves compliance and simplifies treatment regimens, its high acquisition cost makes it unaffordable in regions that rely heavily on out‐of‐pocket payments. As in Japan, annual fees for DPP‐4 inhibitors were reported at $458.7, compared with $273.3 for baseline glucose‐lowering therapies [16]. While in India, SUs continue to dominate as an alternative due to their affordability, despite the higher risks of adverse events such as hypoglycemia and weight gain [22]. The need for cost‐effective yet clinically efficient options in these settings cannot be overemphasized, as financial constraints often lead to treatment interruptions, suboptimal adherence, and ultimately poorer long‐term outcomes [28]. To address these challenges, there is a need to expand access to generic drug options and implement financial assistance programs tailored to resource‐limited healthcare systems.

When DPP‐4/metformin combinations are unaffordable, alternative therapies provide practical and pragmatic solutions. SUs, when used as an add‐on to metformin, are among the most cost‐effective options in resource‐constrained environments. They achieve significant HbA1c reductions, often exceeding those of DPP‐4 inhibitors in the short term, with reductions up to −1.19% reported in several studies [22, 26]. However, their higher risks of hypoglycemia and weight gain are critical drawbacks that necessitate careful monitoring and patient education. For patients with specific needs, particularly those with cardiovascular risks, SGLT‐2 inhibitors or GLP‐1 RAs can offer substantial benefits. SGLT‐2 inhibitors demonstrate significant reductions in cardiovascular events, weight loss, and glycemic control, making them suitable for high‐risk patients when their cost is manageable [28, 33]. Similarly, GLP‐1 RAs provide superior glycemic control with HbA1c reductions of 1.29%–1.51%, along with notable cardiovascular and renal protective effects, although their high cost and injectable form remain barriers to broader use [32, 41]. Expanding subsidies or prioritizing their use in selected high‐risk groups could improve their feasibility in middle‐income countries.

Several limitations have been identified in this review, which must be considered when interpreting the findings. One primary concern is the variability in healthcare costs and cost‐effectiveness thresholds across different countries, which may limit the generalizability of the results. For instance, DPP‐4/metformin combinations are considered cost‐effective in high‐income settings such as Portugal (€9072 per QALY). Still, these findings may not be readily applicable to low‐income regions where cost thresholds are significantly lower [42]. Furthermore, there is limited availability of long‐term adherence data for DPP‐4/metformin combinations. Although short‐term studies reported high adherence rates, such as the 70.3% observed in specific findings [14], it remains unclear whether this adherence can be sustained over time, particularly in low‐income settings where cost and accessibility issues often affect treatment continuity. Lastly, the included studies often reflect healthcare practices and economic models specific to certain regions, which restricts the applicability of findings to diverse populations. Conducting more regionally tailored cost‐effectiveness analyses and exploring innovative pricing strategies could help address these limitations and enhance access to effective diabetes therapies worldwide.

This review identifies the role of DPP‐4/metformin combinations in offering significant clinical benefits, but their high cost poses a major barrier to adoption in low‐income settings. SUs provide a practical, cost‐effective alternative, though their risks must be carefully managed through monitoring and patient education. For patients with specific needs, SGLT‐2 inhibitors and GLP‐1 RAs demonstrate promising benefits but require targeted subsidies or selective prescribing to improve accessibility. Addressing these challenges will require innovative solutions such as broader healthcare subsidies, expanded availability of generic medications, and financial assistance programs tailored to underserved populations.

Future research should focus on evaluating the cost‐effectiveness and long‐term outcomes of alternative metformin combination therapies, especially in low‐income settings with limited resources. Beyond commonly used markers such as HbA1c, it is also essential to consider other indicators that provide a more robust picture of how well these therapies work over time. For example, fibronectin and nitric oxide (NO) are crucial for understanding vascular health and microvascular complications. Elevated fibronectin levels are linked to tissue changes that contribute to diabetic complications like nephropathy and retinopathy. On the other hand, NO plays a key role in endothelial function, and lower NO levels are a sign of endothelial dysfunction, which is common in diabetes. Other markers that would be valuable include vascular endothelial growth factor (VEGF), advanced glycation end products (AGEs), and inflammatory cytokines like interleukin‐6 (IL‐6) and TNF‐*α*. Oxidative stress markers like malondialdehyde (MDA) also help us understand tissue damage at a deeper level. It is also worth including albuminuria for kidney issues, retinal thickness for eye health, and nerve conduction studies for neuropathy. Exploring endothelial function with markers such as endothelin‐1 and asymmetric dimethylarginine (ADMA) would provide further clarity on vascular health outcomes. Finally, future studies should examine how financial and logistical challenges affect long‐term adherence, particularly among low‐income populations. By including these additional markers and tailoring the research to specific regional needs, we can get a clearer, more actionable understanding of how to improve diabetes care in underserved areas.

## Funding

This study was funded by the Northern Border University (Grant NBU‐CRP‐2026‐3770).

## Conflicts of Interest

The authors declare no conflicts of interest.

## Data Availability

The data that support the findings of this study are available from the corresponding author upon reasonable request.
